# Mevalonate Pathway Blockade, Mitochondrial Dysfunction and Autophagy: A Possible Link

**DOI:** 10.3390/ijms160716067

**Published:** 2015-07-15

**Authors:** Paola Maura Tricarico, Sergio Crovella, Fulvio Celsi

**Affiliations:** 1 Department of Medicine, Surgery and Health Sciences, University of Trieste, Piazzale Europa 1, 34128 Trieste, Italy; E-Mail: sergio.crovella@burlo.trieste.it; 2 Institute for Maternal and Child Health “Burlo Garofolo”, via dell’Istria 65/1, 34137 Trieste, Italy; E-Mail: fulvio.celsi@gmail.com

**Keywords:** autophagy, mevalonate pathway, mitochondrial dysfunction, inflammation, Mevalonate Kinase Deficiency, statins

## Abstract

The mevalonate pathway, crucial for cholesterol synthesis, plays a key role in multiple cellular processes. Deregulation of this pathway is also correlated with diminished protein prenylation, an important post-translational modification necessary to localize certain proteins, such as small GTPases, to membranes. Mevalonate pathway blockade has been linked to mitochondrial dysfunction: especially involving lower mitochondrial membrane potential and increased release of pro-apoptotic factors in cytosol. Furthermore a severe reduction of protein prenylation has also been associated with defective autophagy, possibly causing inflammasome activation and subsequent cell death. So, it is tempting to hypothesize a mechanism in which defective autophagy fails to remove damaged mitochondria, resulting in increased cell death. This mechanism could play a significant role in Mevalonate Kinase Deficiency, an autoinflammatory disease characterized by a defect in Mevalonate Kinase, a key enzyme of the mevalonate pathway. Patients carrying mutations in the *MVK* gene, encoding this enzyme, show increased inflammation and lower protein prenylation levels. This review aims at analysing the correlation between mevalonate pathway defects, mitochondrial dysfunction and defective autophagy, as well as inflammation, using Mevalonate Kinase Deficiency as a model to clarify the current pathogenetic hypothesis as the basis of the disease.

## 1. Mevalonate Pathway

The mevalonate pathway, fundamental for cholesterol synthesis, is one of the most important metabolic networks in the cell; it provides essential cell constituents, such as cholesterol, and some of its branches produce key metabolites, such as geranylgeranyl pyrophosphate and farnesyl pyrophosphate, necessary for normal cell metabolism.

**Figure 1:**
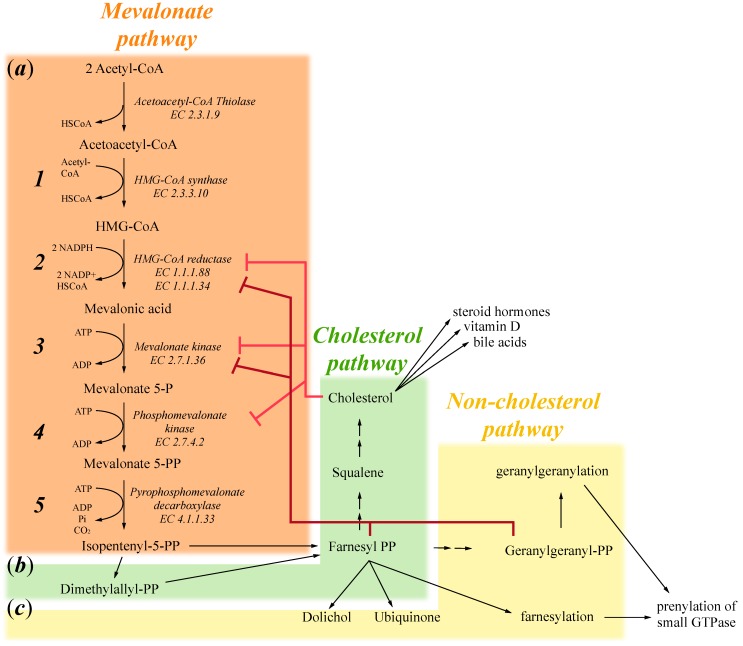
Schematic representation of the mevalonate pathway divided into: (**a**) The mevalonate pathway that produces mevalonate 5-PP and then isppententenyl 5-PP; (**b**) The cholesterol pathway that produces cholesterol, which in turn induces the formation of steroid hormones, vitamin D and bile acids; and (**c**) The non-cholesterol pathway important for the production of farnesyl-PP and geranylgeranyl-PP that induces respectively farnesylation and geranylgeranylation of small GTPase.

**Figure 2:**
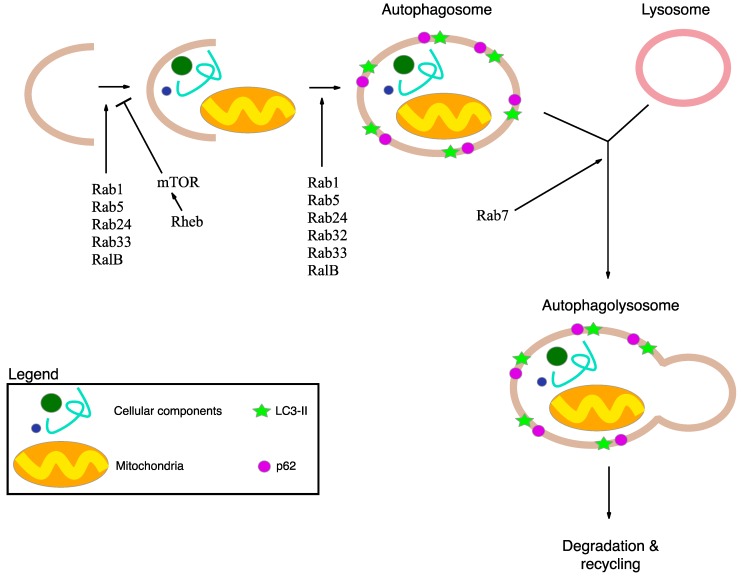
Schematic representation of macroautophagy mechanism and its main actors. Macroautophagy delivers cellular components and damaged or redundant organelles (such as mitochondria), to the lysosome through the intermediary of a double membrane-bound vesicle, referred to as an autophagosome. Autophagy is initiated by the formation of the isolation membrane that induces the formation of autophagosome. Subsequently, the autophagosome fuses with the lysosome to form an autolysosome. Finally all the material is degraded in the autophagolysosome and recycled. The p62 protein interacts with damaged proteins in the cells, and the complexes are then selectively tied to the autophagosome through LC3-II. Rabs, Rheb and RalB are autophagy-related protein important for the regulation of macroautophagy mechanism.

The first step of the mevalonate pathway is the synthesis of 3-hydroxy-3-methylglutaryl-CoA (HMG-CoA) from three molecules of acetyl-CoA, firstly by a condensation reaction forming acetoacetyl-CoA through acetoacetyl-CoA thiolase (EC 2.3.1.9) and subsequently through a second condensation between acetoacetyl-CoA and a third acetyl-CoA molecule catalysed by HMG-CoA synthase (EC 2.3.3.10) (Figure 1a, 1). In the second step, HMG-CoA is reduced to mevalonate acid by NADPH, a reaction catalysed by the HMG-CoA reductase (HMGR) enzyme (EC 1.1.1.88 and EC 1.1.1.34). HMGR is the rate-limiting enzyme for the mevalonate pathway and is one of the most finely regulated enzymes [1]. Regulation begins at the transcriptional level; if cholesterol or other sterol isoprenoids are in shortage, sterol regulatory element binding proteins (SREBP) are activated and they bind to sterol regulatory elements (SREs) present on the HMGR promoter, increasing its transcription [2,3]. Cholesterol also regulates the degradation of HMGR, promoting its association with gp78, an ubiquitin-E3 ligase that directs the enzyme towards proteasome 26s. HMGR is also regulated at the post-translational level, by phosphorylation mediated through AMP-activated protein kinase (AMPK). This enzyme is sensitive to the AMP:ATP ratio, and is activated by increased AMP concentration, thus in casea of metabolic stress, it deactivates HMGR, reducing cellular metabolism [4] (Figure 1a, 2).

**Figure 3:**
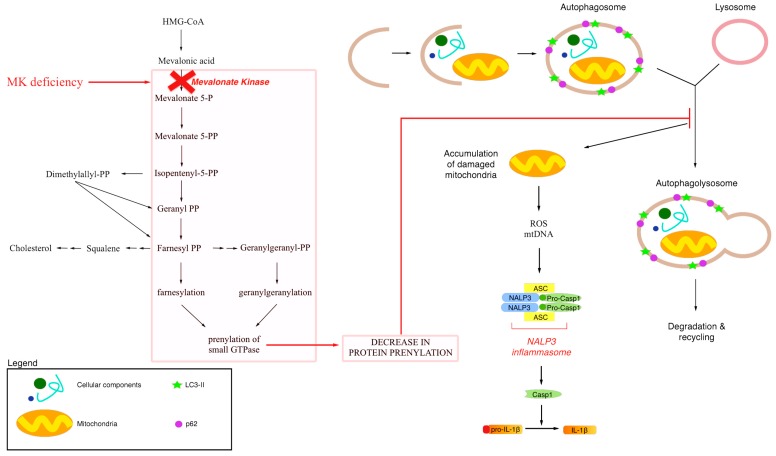
Schematic representation of a possibile link between defective protein prenylation, mitochondrial dysfunction and autophagy. Mevalonate Kinase Deficiency is characterized by a block of the mevalonate pathway induces by mutation in a gene that encodes for Mevalonate Kinase. Blockade of the mevalonate pathway induces decrease in protein prenylation that could alter the macroautophagy mechanism and in particular mitochondrial degradation and recycling. Accumulation of damaged mitochondria induces ROS production and mtDNA release. All these events are important for the activation of NALP3 inflammasome that cleaves and activates IL-1b.

The third key enzyme of the mevalonate pathway is the one responsible for converting mevalonic acid into mevalonate-5-phosphate, a key pathway intermediate. Mevalonate kinase (EC 2.7.1.36) (MVK) catalyses this conversion, using ATP as a phosphate donor and energy source. Furthermore, this enzyme is finely regulated, firstly at transcriptional level in a similar manner of HMGR: SREs are present at the MVK promoter and increases its transcription upon cholesterol shortage [5]. In addition, MVK presents feedback inhibition from some of the mevalonate pathway substrates, specifically geranylgeranyl pyrophosphate and farnesyl pyrophosphate, demonstrating that non-sterol isoprenoid could have a key role in regulation of this enzyme [6] (Figure 1a, 3).

In the fourth step of the mevalonate pathway, Mevalonate-5-phosphate is then converted into mevalonate-5-pyrophosphate by phosphomevalonate kinase (EC 2.7.4.2), using again ATP as phosphate and energy donor. Differently from MVK, this enzyme does not present feedback inhibition from its products [6]. However, various compounds were recently found to be inhibitors for this enzyme, suggesting novel mechanisms to inhibit the mevalonate pathway [7] and more interestingly, phosphomevalonate kinase appears also to be regulated by cholesterol shortage, as for mevalonate kinase and HMGR [8]. Indeed, cholesterol shortage induces increases in all the three first enzymes of the mevalonate pathway, thus guaranteeing a continued supply of this key membrane component (Figure 1a, 4).

The fifth enzyme of the mevalonate pathway is pyrophosphomevalonate decarboxylase or diphosphomevalonate decarboxylase (EC 4.1.1.33). It converts mevalonate-5-pyrophosphate into isopentenyl-5-pyrophosphate (IPP), the final product of mevalonate pathway and the starting substrate for successive biosyntheticals reactions, especially cholesterol and isoprenoid production. This enzyme performs two key reactions: firstly, it phosphorylates mevalonate-5-pyrophosphate generating an intermediate product that, secondly, it is dephosphorylated and decarboxylated, obtaining thus IPP as a final product (Figure 1a, 5).

The mevalonate pathway, as described above, generates a key intermediate for cholesterol production, a fundamental constituent of cell membranes. Moreover, cholesterol is also converted to steroid hormones, regulating different cellular pathways, vitamin D and bile acid production (Figure 1b). IPP is the first step also in other, non-cholesterol, reactions; it is important for the production of farnesyl-pyrophosphate (FPP). FPP is converted in dolichols, used to assemble carbohydrate chains in glycoproteins, or in ubiquinones (or coenzyme Q10), electron transporters in mitochondria; or it is used to farnesylate or geranylate proteins, thus targeting them to cell membranes (Figure 1c). In summary, the mevalonate pathway is responsible for numerous cellular processes and the key enzymes described above undergo different regulation to maintain a constant supply of IPP.

## 2. Exogenous Mevalonate Pathway Blockade

The principal compounds that induce exogenous mevalonate pathway blockade are the statins, which are a class of compounds that act as competitive inhibitors of 3-hydroxy-3-methylglutaryl coenzyme-A (HMG-CoA) reductase, a key enzyme of the mevalonate pathway, which converts HMG-CoA into mevalonic acid. Statins, in general, are able to bind to a portion of HMG-CoA binding site, thus blocking the access of this substrate to the active site of the enzyme; effectively reducing the rate of mevalonate productions [9,10].

In 1976 Endo and coauthors discovered the first statin, isolated from *Penicillium citrinium* [11]. Subsequently, over the last two decades, several statins have been identified and classified in several ways. The most commonly used classification divides them into statins produced by fungi (such as Lovastatin, Simvastatin) and statins synthetically made (such as Atorvastatin, Fluvastatin).

All statins share a conserved HMG-like moiety covalently linked to a more or less extended hydrophobic group.

By blocking HMG-CoA reductase, statins induce a decrease in cholesterol level and simultaneously other by-products of the mevalonate pathway such as farnesyl pyrophosphate (FPP), geranylgeranyl pyrophosphate (GGPP), dolichols and coenzyme Q10 [12,13]. As reviewed in Winter-Vann and Casey (2005), inhibition of HMG-CoA reductase has a pleiotropic effect, due to the different affinities of key enzymes in the mevalonate pathway. FPP, the main metabolite in this pathway, could be converted to cholesterol through squalene synthase and this enzyme has a Km for the substrate of about 2 μM. GGPP synthase, instead, could convert FPP to GGPP, with a Km of 1 μM; GGPP is attached to different proteins (the majority of which pertain to the Rab family) to ensure their correct localization. On the other hand, protein farnesyl trasferase (FTase) uses FPP to attach a farnesyl group to specific proteins, such as the family of small GTPase proteins (Ras and Rho GTPases), with a Km of 5 nM. Therefore, inhibition of HMG-CoA reductase lowers FPP levels and the first consequence is a reduction in cholesterol levels; following that, GGPP levels are reduced, causing mislocalization and loss of activity of specific proteins. Instead, due to the high affinity of FTase towards FPP, farnesylation levels of key cellular enzymes remain stable [14].

Indeed, a widely adopted view considers the pleiotropic effects of statins independent of lowering cholesterol levels, but rather connected to a lack of these prenylated proteins [12]. In the last few years there has been an increase in interest of these pleiotropic effects, because of their possible main responsibility for statin anti-cancer and immunomodulatory effects [15,16,17,18].

For all these reasons, the role of statins are debatable, and there are many studies describing statins as drugs for treatment of a variety of disease such as hypercholesterolemia, cancer, cardiovascular diseases, inflammatory diseases [19,20,21,22,23,24].

Furthermore, statins are used as a pharmacological compound to biochemically reproduce some features of Mevalonate Kinase Deficiency (MKD)—a pathology characterized by a defect in a key enzyme of mevalonate pathway [13,25,26]. In some studies, mevalonate pathway blockade, obtained in neuronal and monocytic cell lines by statin (Lovastatin) administration, induces an increase of apoptosis correlated to mitochondrial damage [27,28,29].

Also, Van der Burgh and co-workers have recently demonstrated that mevalonate pathway blockade, obtained in monocytic cell line by statin (Simvastatin) administration, produces mitochondrial damage and autophagy impairment, related to a decrease in protein prenylation levels [25,30].

### 2.1. Mitochondrial Dysfunction and Statin

Mevalonate pathway blockade, obtained by treatment with statins, has been linked to mitochondrial dysfunction, specifically by lowering mitochondrial membrane potential and increasing release of pro-apoptotic factors.

Usually, mitochondrial dysfunction is associated with intrinsic apoptosis, also known as the mitochondrial apoptotic pathway. This pathway is characterized by activation of caspase-9 and -3, and inhibition or activation of anti- or pro-apoptotic Bcl-2 family members. Furthermore, mitochondrial membrane potential decreases, causing release of pro-apoptotic factors, oxidative stress and then cell death [31].

In a biochemical MKD model, obtained by Lovastatin treatment in neuroblastoma cell lines, we observed mitochondrial dysfunction correlated to increased intrinsic apoptosis, also confirmed by activation of caspase-3 and -9 [27,28]; furthermore, in monocyte cell lines, we observed a similar increase in oxidative stress [29].

Mitochondrial dysfunction, caused by statins, could be related to oxidative stress, shortage of prenylated proteins or both. In fact, it was observed that the block of mevalonate pathway, obtained by statin (Simvastin) treatment in endothelial cancer cell lines, resulted in G1 cell cycle arrest, apoptosis, DNA damage and cellular stress [32].

Another study showed that simvastatin, in lung cancer cells, inhibited the proliferation and significantly increased oxidative stress, in particular augmenting reactive oxygen species (ROS) production and the activity of total superoxide dismutase (SOD) and in particular the mitochondrial form, superoxide dismutase 2 (SOD2) [33].

Strong oxidative stress, which induces mitochondrial dysfunction, could be due to the action of statins on the mevalonate pathway, decreasing coenzyme Q10 and dolichol levels, considered as anti-oxidants defense systems.

Coenzyme Q10 is a product of the mevalonate pathway and is an important electron transporter of the mitochondrial respiratory chain. A decrease in coenzyme Q10 levels, caused by mevalonate pathway blockade, could result in an abnormal mitochondrial respiratory function causing mitochondrial and oxidative damage [34].

Dolichol, a polyprenol compound, is an important free-radical scavenger in cell membranes [35]. Ciosek and co-workers observed a significant decrease in dolichol levels after Lovastatin administration in *in vivo* models [36]; a lack of this compound might cause oxidative stress and mitochondrial damage [13,37].

Nevertheless, mitochondrial dysfunction caused by statins could also be related to a decrease in prenylated protein levels; indeed, statins treatment could lead to a reduction in cholesterol level, and also in farnesyl pyrophosphate (FPP) and in geranylgeranyl pyrophosphate (GGPP). Xia and co-workers have demonstrated that apoptosis induced by Lovastatin treatment, in human AML cells is connected to a decrease in GGPP and, to a lesser extent, related to a FPP decrease [38].

Agarwal and co-workers also observed the close correlation between decrease in prenylated proteins levels, apoptosis and mitochondrial damage in Lovastatin-treated colon cancer cells. The treatment caused a decrease in expression of anti-apoptotic protein Bcl-2 and an increase of pro-apoptotic protein such as Bax; the subsequent addition of GGPP prevented Lovastatin apoptosis, confirming a key role of prenylated proteins levels [39].

Recently, Van der Burgh and co-workers have demonstrated that, in simvastatin-treated cells, mitochondria clearance is reduced, with lower oxygen consumption and glycolysis rate. These conditions suggested that accumulation of damaged mitochondria could be the trigger for NACHT, LRR and PYD domains-containing protein 3 (NALP3) inflammasome activation. The authors also speculate that prenylated proteins could be the main mediators of statin adverse effects [25,30].

Further confirmations that statins treatment could impair mitochondrial activity come from two recent studies done using a completely different model, *C. elegans*. In the first paper, the authors show that animals resistant to statin treatment have an increased mitochondrial unfolded protein response (UPRmt) which, they speculate, could lower protein turnover and thus lessening the need for protein prenylation [40]. In the second paper, the authors demonstrate that statin abrogates the *C. elegans* ability to sense mitochondrial damage and that this ability could be partially rescued through GGPP subministration [41]. Taken together, these results demonstrate once more the key role of protein prenylation in mitochondrial homeostasis.

A mitochondrial-specific effect of statin, probably mediated through lowering prenylated proteins, suggests that this class of compound could be considered as anti-cancer drugs. However, further studies are necessary to completely clarify the variety of effects of these drugs and at present, only hypotheses have been raised to explain actions of statins on cellular survival.

### 2.2. Autophagy and Statins

Mevalonate pathway blockade has been linked to defective autophagy, possibly causing inflammasome activation and subsequent cell death. Autophagy (macroautophagy) is the main catabolic mechanism involved in the turnover of cytoplasmic components and selective removal of damaged or redundant organelles (such as mitochondria, peroxisomes and endoplasmic reticulum), through the lysosome machinery. Initial steps include the formation of phagophore or pre-autophagosome, an isolated membrane able to elongate and forming the autophagosome, a double-membrane compartment that sequesters the cytoplasmic materials. Subsequently, the fusion of autophagosome with lysosome forms the autolysosome, where the captured material is degraded [42] (Figure 2).

Autophagy involves numerous molecular mediators called autophagy-related (ATG) proteins; among these, prenylation of proteins appears to be one of the key regulation mechanisms [43]. Specifically, different small GTPase are indicated as ATG proteins: Rabs, Rheb, RalB. Rabs are crucial proteins for developing subdomains on membranes to facilitate maturation; recent studies have shown that some Rabs are essential for autophagy. The best known Rabs at the present time are: Rab1, able to regulate the autophagosome formation; Rab5, an early endosome protein, responsible for autophagosome membrane elongation and autophagosome formation, regulating Beclin1-Vps34-Atg6 class III PI3-kinase complex; Rab7, a late endosome protein, responsible for autophagosome maturation, promoting the microtubule plus-end-directed transport and facilitating fusion of autophagosome with lysosome; Rab24, normally present in reticular distribution around the nucleus, is important for autophagosome maturation; Rab32, generally present in mitochondria, regulates the membrane trafficking, and is required for autophagosome formation; Rab33 modulates autophagosome formation [44,45] (Figure 2).

Ras homology enriched in brain (Rheb) directly binds and selectively activates the multiprotein complex 1 of mammalian target of rapamycin (mTORC1) [46] which is composed of mTOR, a negative regulator of autophagy, and mLST8 [47,48]. mTOR activity inhibits mammalian autophagy and indeed a recent study demonstrated that autophagy impairment is correlated to mTORC1 hyperactivation in β-cell [49] (Figure 2).

RalB localizes in nascent autophagosome and is activated due to nutrient deprivation and, thanks to the binding to its effector Exo84, induces the assembly of active Beclin1-VPS34 and ULK1. The resulting complex induces the isolation of pre-autophagosomal membrane and maturation of autophagosomes [50,51] (Figure 2).

All these proteins require prenylation for their activation, either farnesylated or geranylgeranylated; indeed statins, blocking the mevalonate pathway and causing a decrease in prenylated proteins levels, could play a regulatory role in autophagy. Nevertheless, the statin effects in autophagy remain poorly understood.

Recently, Van der Burgh and co-workers have observed defective autophagy in statin-treated monocytes, correlated to damaged mitochondria and NALP3 inflammasome activation [25]. The same authors have subsequently demonstrated that statin treatment increases levels of unprenylated RhoA, which in turn activates protein kinase B (PKB) possibly playing a role in statin-induced autophagy blockade [30].

On the contrary, other studies show that treatment with statins induces an increase in autophagy levels, and for this reason, statins could be considered as anti-cancer drugs. Indeed, a study showed that statins, such as Cerivastatin, Pitavastatin and Fluvastatin, are the most potent autophagy inducing agents in human cancer cells; the authors, however, did not analyze levels of p62, thus the possibility that autophagy is increased but subsequentrly blocked was not examined [52].

Another study demonstrated that statin induces autophagy through depleting cellular levels of geranylgeranyl diphosphonate (GGPP), independently of the decreased activity in two major small G proteins, Rheb and Ras [53]. However, the authors did not examine all autophagic pathways, thus the observations are not conclusive and further investigations are needed.

Lastly, Wei and co-workers observed that simvastatin inhibits the Rac1-mTOR pathway and thereby increases autophagy, in coronary arterial myocytes [54].

All these results show that the role of statin in autophagy is related to GGPP and prenylated proteins levels, thus being important actors in this mechanism. Indeed, cellular differences in GGPP and prenylated proteins levels could explain the contradictory findings in the studies described above. However, further studies are necessary to clarify the mechanism of action and the molecular targets involved in statin-modulated autophagy.

## 3. Endogenous Mevalonate Pathway Blockade

The mevalonate pathway could also be blocked by enzymatic defects due to mutations in genes involved in this pathway. In particular, a rare disease involving mutations on *MVK* gene (12q24.11) has been described: Mevalonate Kinase Deficiency (MKD). Currently, 82 mutations of the *MVK* gene have been reported in the Human Gene Mutation Database [55].

MKD possesses two distinct phenotypes: a milder one, also called Hyper IgD Syndrome (HIDS; OMIM#260920), in which the patients suffer recurrent fevers, have skin rashes, hepatosplenomegalia and generally a sustained inflammatory response; and a severe, rarer, one, called Mevalonic Aciduria (MA; OMIM #610377), characterized by the involvement of the Central Nervous System, with cerebellar ataxia, psychomotor retardation and also, as in HIDS, recurrent fever attacks [56].

Residual MVK enzymatic activity marks the boundary between HIDS and MA, with MA patients having less than 1% activity, while HIDS between 1% and 7% of activity [57]. Initially, disease pathogenesis was thought to derive from low cholesterol levels, being MVK a central enzyme in the mevalonate pathway. However, patients, either with HIDS or MA, showed normal cholesterol levels, probably due to dietary intake. Subsequently, accumulation of Mevalonic acid was indicated as responsible for the MKD phenotype. Still, a small clinical trial, involving two MA patients, using statin (Lovastatin) to reduce Mevalonic acid, resulted in worsening of the symptoms [58]; on the contrary, Simvastatin appeared to be beneficial for treating HIDS patients [56]. Thus, the hypothesis pointing to Mevalonic acid levels as causative for MKD does not explain completely the disease’s manifestations. Celec and Behuliak in 2008 hypothesized that MVK dysfunctions could diminish non-steroid isoprenoids, causing oxidative stress and ultimately leading to chronic hyperinflammation [13].

The shortage of isoprenoids, correlated to a severe reduction in protein prenylation, in particular of geranylgeranyl pyrophosphate (GGPP), has been linked with the activation of caspase-1 and thereby with the production of IL-1β [59,60].

In particular, the IL-1 family is strongly supposed to play a fundamental role in MKD inflammatory processes, indeed, several biological therapies have successfully targeted these molecules [61,62,63].

In a previous work it has been shown that, in monocytes from MKD patients, a key component of the inflammation machinery is NACHT, LRR and PYD domains-containing protein 3 (NALP3) [64]; NALP3 interacts with another protein, pyrin domain (PYD) of apoptosis-associated speck-like protein containing a CARD domain (ASC), constituting the inflammasome platform. The CARD domain recruits pro-caspase-1, which self-cleaves into active caspase-1 and then converts pro-IL-1β to active IL-1β, activating one of the main pathways of inflammation [65,66].

However it remains an open question how isoprenoid shortage, as in MKD and in presence of the biochemical block, activates NALP3 and the inflammasome pathway.

Recently Van der Burgh and co-workers have demonstrated that, in MKD patients’ cells and in statin-treated monocytes, autophagy is impaired and specifically mitochondria clearance is slowed, suggesting that accumulation of damaged mitochondria could be the trigger to NALP3 inflammasome activation [25]. The same group has recently reported that statin treatment increases levels of unprenylated Ras homolog gene family member A (RhoA), which in turn activates PKB, representing then the hypothetical starting point for autophagy. The authors also demonstrated that levels of unprenylated RhoA correlate with IL-1b release, thus partially confirming this hypothetical link [25,30].

## 4. Autophagy, Inflammation and Damaged Mitochondria

An emerging concept in recent years tightly associates autophagy with inflammation regulation mechanisms. Autophagy can regulate different aspects of the immune response: it is involved in the degradation of bacteria/virus engulfed by the cell, it regulates Pattern Recognition Receptors (PRRs) and can act as their effector, it can process Antigens for MHC presentation and finally autophagy can regulate inflammasome activation and secretion of different cytokines [67]. The first three mechanisms have been extensively described by Deretic (2013) [68], and will be briefly discussed here.

Autophagy can be envisaged as a mechanism to clear the cytosol from invading intracellular pathogens, either bacteria or viruses, which are engulfed in autophagic membranes and targeted to lysosomes to be degraded. This process is facilitated by sequestosome 1/p62-like receptors (SLRs) that recognize pathogens and facilitate their encasement in autophagosomes, possessing an LC3 interacting region.

Furthermore, autophagy could be an effector for PRRs, degrading targets marked by toll-like receptors (TLRs) or it can help in delivering ligands to TLRs, as for TLR7 [68].

Antigen processing for MHC II presentation represents another important mechanism regulated by autophagy. Indeed, autophagy is crucial for viral immunosurvelliance and is also inhibited by HIV-1 by upregulating mTOR in dendritic cells [69,70]. Moreover, autophagy is required for positive selection of naïve T cells in thymus, whereas knocking-out key autophagy proteins results in autoimmune syndromes [71].

How autophagy regulates inflammasome activation is currently a subject of numerous studies and a general consensus has not been yet reached. However, two hypotheses are at the moment explored: a first one suggesting that autophagy regulates processing of IL-1b and other pro-inflammatory molecules; the second proposing that autophagy removes damaged mitochondria, thus dampening NALP3 activation. It is possible that those two processes are not mutually exclusive and act in parallel to maintain the inflammatory status in “inactive” condition.

Direct regulation of IL-1b processing by inflammasomes has been demonstrated in macrophages and *in vivo* where treatment with rapamycin (autophagy inducer) decreases its secreted and circulating levels [72]. Further confirmation came from the work of Shi and co-authors (2012), in which they demonstrate how inflammasome activation induces autophagy and autophagosomes formation containing inflammasomes components such as ASC or absent in melanoma 2 (AIM2), in a self-limiting process. IL-1b does not possess a “canonical” secretory signature and it is translated in the cytosol, via polyribosomes linked to the cytoskeleton [73,74,75]. Later it was demonstrated that secretion of IL-1b is dependent on its localization in lysosome-associated vesicles and agents that regulates autophagy can modulate its release [72,76]. These works clearly show the regulation of IL-1b processing by autophagy.

Other studies put autophagy upstream of inflammasome activation. Reactive oxygen species (ROS) can activate NALP3 inflammasome and damaged mitochondria are the main source for ROS. Mitophagy, a specialized form of autophagy, constantly removes damaged mitochondria, thus lowering ROS levels. If cells are challenged with 3-methyladenine, a blocker of mitophagy, NALP3 activation is increased and redistributed near mitochondria-ER contact points, working as a sensor for mitochondrial damage [77].

Moreover, mitochondrial DNA (mtDNA) can work as NALP3 activation inducer, thus sensing mitochondrial damage and 3-MA could increase NALP3 activation caused by mtDNA [78,79]. These data show a possible pipeline: decreased mitophagy leading to increased ROS and mtDNA in cytosol, leading to increased NALP3 activation.

The two mechanisms described above are not necessarily self-exclusive. It is then possible that autophagy machinery collaborates in dampening inflammation and a disturbance in mitochondria homeostasis could lead to exacerbating inflammation.

In MKD is it then possible that a disturbance in autophagy mechanisms causes damage in mitochondria, impairing their recycling and thus increasing ROS levels; this, in turn, increases activation of inflammation. This chain of events still remains to be verified; however exploring this mechanism could represent a novel strategy to fight this debilitating disease.

## 5. Conclusive Remarks

MKD is an orphan drug disease, so many efforts are being made in search of potential targets for novel treatments tailored to prevent, or at least to diminish, apoptosis in MKD patients. Several studies reported *in vitro* administration of natural and synthetic isoprenoids to restore the mevalonate pathway in cell cultures (both models and patients’ derived monocytes) treated with statins to biochemically mimic the genetic defect.

As described above, a possible link exists between defective protein prenylation and mitochondrial dysfunction, supposedly made by autophagy (Figure 3).

Furthermore, it remains to be determined how autophagy is impaired in MKD. Protein prenylation seems to be one of the regulation mechanisms involved in autophagy. Compounds able to restore protein prenylation could be considered as potential therapy to tackle MKD; unfortunatly such compounds are at the present time, not actively researched. Other strategies, such as modulation of farnesyl protein transferase could be exploited to fight MKD.

For all these reasons, our review aimed at recalling the attention of the scientific community on another possible mechanism at the basis of MKD pathogenesis, and intends to point out that autophagy should be considered when trying to design novel therapeutic strategies to fight cell death in MKD patients.

## References

[1] Goldstein J.L., Brown M.S. (1990). Regulation of the mevalonate pathway. Nature.

[2] Horton J.D. (2002). Sterol regulatory element-binding proteins: Transcriptional activators of lipid synthesis. Biochem. Soc. Trans..

[3] Weber L.W., Boll M., Stampfl A. (2004). Maintaining cholesterol homeostasis: Sterol regulatory element-binding proteins. World J. Gastroenterol..

[4] Burg J.S., Espenshade P.J. (2011). Regulation of HMG-CoA reductase in mammals and yeast. Progress Lipid Res..

[5] Murphy C., Murray A.M., Meaney S., Gåfvels M. (2007). Regulation by SREBP-2 defines a potential link between isoprenoid and adenosylcobalamin metabolism. Biochem. Biophys. Res. Commun..

[6] Hinson D.D., Chambliss K.L., Toth M.J., Tanaka R.D., Gibson K.M. (1997). Post-translational regulation of mevalonate kinase by intermediates of the cholesterol and nonsterol isoprene biosynthetic pathways. J. Lipid Res..

[7] Boonsri P., Neumann T.S., Olson A.L., Cai S., Herdendorf T.J., Miziorko H.M., Hannongbua S., Sem D.S. (2013). Molecular docking and NMR binding studies to identify novel inhibitors of human phosphomevalonate kinase. Biochem. Biophys. Res. Commun..

[8] Olivier L.M., Chambliss K.L., Gibson K.M., Krisans S.K. (1999). Characterization of phosphomevalonate kinase: Chromosomal localization, regulation, and subcellular targeting. J. Lipid Res..

[9] Istvan E.S., Deisenhofer J. (2001). Structural mechanism for statin inhibition of HMG-CoA reductase. Science.

[10] Corsini A., Maggi F.M., Catapano A.L. (1995). Pharmacology of competitive inhibitors of HMG-CoA reductase. Pharmacol. Res..

[11] Endo A., Kuroda M., Tsujita Y. (1976). ML-236A, ML-236B, and ML-236C, new inhibitors of cholesterogenesis produced by Penicillium citrinium. J. Antibiot. (Tokyo).

[12] Alegret M., Silvestre J.S. (2006). Pleiotropic effects of statins and related pharmacological experimental approaches. Methods Find Exp. Clin. Pharmacol..

[13] Celec P., Behuliak M. (2008). The lack of non-steroid isoprenoids causes oxidative stress in patients with mevalonic aciduria. Med. Hypotheses.

[14] Winter-Vann A.M., Casey P.J. (2005). Post-prenylation-processing enzymes as new targets in oncogenesis. Nat. Rev. Cancer.

[15] Wong W.W., Dimitroulakos J., Minden M.D., Penn L.Z. (2002). HMG-CoA reductase inhibitors and the malignant cell: The statin family of drugs as triggers of tumor-specific apoptosis. Leukemia.

[16] Sassano A., Platanias L.C. (2008). Statins in tumor suppression. Cancer Lett..

[17] Osmak M. (2012). Statins and cancer: Current and future prospects. Cancer Lett..

[18] Blum A., Shamburek R. (2009). The pleiotropic effects of statins on endothelial function, vascular inflammation, immunomodulation and thrombogenesis. Atherosclerosis.

[19] Olsson A.G., Istad H., Luurila O., Ose L., Stender S., Tuomilehto J., Wiklund O., Southworth H., Pears J., Wilpshaar J.W. (2002). Effects of rosuvastatin and atorvastatin compared over 52 weeks of treatment in patients with hypercholesterolemia. Am. Heart J..

[20] Cardwell C.R., Mc Menamin Ú., Hughes C.M., Murray L.J. (2015). Statin use and survival from lung cancer: A population-based cohort study. Cancer Epidemiol. Biomarkers Prev..

[21] Shepherd J., Blauw G.J., Murphy M.B., Bollen E.L., Buckley B.M., Cobbe S.M., Ford I., Gaw A., Hyland M., Jukema J.W., PROSPER study group (2002). Pravastatin in elderly individuals at risk of vascular disease (PROSPER): A randomised controlled trial. Lancet.

[22] Dursun S., Çuhadar S., Köseoğlu M., Atay A., Aktaş S.G. (2014). The anti-inflammatory and antioxidant effects of pravastatin and nebivolol in rat aorta. Anadolu Kardiyol. Derg..

[23] Leung B.P., Sattar N., Crilly A., Prach M., McCarey D.W., Payne H., Madhok R., Campbell C., Gracie J.A., Liew F.Y. (2003). A novel anti-inflammatory role for simvastatin in inflammatory arthritis. J. Immunol..

[24] Barsante M.M., Roffê E., Yokoro C.M., Tafuri W.L., Souza D.G., Pinho V., Castro M.S., Teixeira M.M. (2005). Anti-inflammatory and analgesic effects of atorvastatin in a rat model of adjuvant-induced arthritis. Eur. J. Pharmacol..

[25] Van der Burgh R., Nijhuis L., Pervolaraki K., Compeer E., Jongeneel L.H., van Gijn M., Coffer P.J., Murphy M.P., Mastroberardino P.G., Frenkel J. (2014). Defects in mitochondrial clearance predispose human monocytes to interleukin-1β hypersecretion. J. Biol. Chem..

[26] Kuijk L.M., Beekman J.M., Koster J., Waterham H.R., Frenkel J., Coffer P.J. (2008). HMG-CoA reductase inhibition induces IL-1β release through Rac1/PI3K/PKB-dependent caspase-1 activation. Blood.

[27] Marcuzzi A., Tricarico P.M., Piscianz E., Kleiner G., Brumatti L.V., Crovella S. (2013). Lovastatin induces apoptosis through the mitochondrial pathway in an undifferentiated SH-SY5Y neuroblastoma cell line. Cell Death Dis..

[28] Marcuzzi A., Zanin V., Piscianz E., Tricarico P.M., Vuch J., Girardelli M., Monasta L., Bianco A.M., Crovella S. (2012). Lovastatin-induced apoptosis is modulated by geranylgeraniol in a neuroblastoma cell line. Int. J. Dev. Neurosci..

[29] Tricarico P.M., Kleiner G., Valencic E., Campisciano G., Girardelli M., Crovella S., Knowles A., Marcuzzi A. (2014). Block of the mevalonate pathway triggers oxidative and inflammatory molecular mechanisms modulated by exogenous isoprenoid compounds. Int. J. Mol. Sci..

[30] Van der Burgh R., Pervolaraki K., Turkenburg M., Waterham H.R., Frenkel J., Boes M. (2014). Unprenylated RhoA contributes to IL-1β hypersecretion in mevalonate kinase deficiency model through stimulation of Rac1 activity. J. Biol. Chem..

[31] Tricarico P.M., Marcuzzi A., Piscianz E., Monasta L., Crovella S., Kleiner G. (2013). Mevalonate kinase deficiency and neuroinflammation: Balance between apoptosis and pyroptosis. Int. J. Mol. Sci..

[32] Schointuch M.N., Gilliam T.P., Stine J.E., Han X., Zhou C., Gehrig P.A., Kim K., Bae-Jump V.L. (2014). Simvastatin, an HMG-CoA reductase inhibitor, exhibits anti-metastatic and anti-tumorigenic effects in endometrial cancer. Gynecol. Oncol..

[33] Li Y., Fu J., Yuan X., Hu C. (2014). Simvastatin inhibits the proliferation of A549 lung cancer cells through oxidative stress and up-regulation of SOD2. Pharmazie.

[34] Tavintharan S., Ong C.N., Jeyaseelan K., Sivakumar M., Lim S.C., Sum C.F. (2007). Reduced mitochondrial coenzyme Q10 levels in HepG2 cells treated with high-dose simvastatin: A possible role in statin-induced hepatotoxicity?. Toxicol. Appl. Pharmacol..

[35] Bergamini E., Bizzarri R., Cavallini G., Cerbai B., Chiellini E., Donati A., Gori Z., Manfrini A., Parentini I., Signori F. (2004). Ageing and oxidative stress: A role for dolichol in the antioxidant machinery of cell membranes?. J. Alzheimers Dis..

[36] Ciosek C.P., Magnin D.R., Harrity T.W., Logan J.V., Dickson J.K., Gordon E.M., Hamilton K.A., Jolibois K.G., Kunselman L.K., Lawrence R.M. (1993). Lipophilic 1,1-bisphosphonates are potent squalene synthase inhibitors and orally active cholesterol lowering agents *in vivo*. J. Biol. Chem..

[37] Sirvent P., Mercier J., Lacampagne A. (2008). New insights into mechanisms of statin-associated myotoxicity. Curr. Opin. Pharmacol..

[38] Xia Z., Tan M.M., Wong W.W., Dimitroulakos J., Minden M.D., Penn L.Z. (2001). Blocking protein geranylgeranylation is essential for lovastatin-induced apoptosis of human acute myeloid leukemia cells. Leukemia (Baltimore).

[39] Agarwal B., Bhendwal S., Halmos B., Moss S.F., Ramey W.G., Holt P.R. (1999). Lovastatin augments apoptosis induced by chemotherapeutic agents in colon cancer cells. Clin. Cancer Res..

[40] Rauthan M., Ranji P., Aguilera Pradenas N., Pitot C., Pilon M. (2013). The mitochondrial unfolded protein response activator ATFS-1 protects cells from inhibition of the mevalonate pathway. Proc. Natl. Acad. Sci. USA.

[41] Liu Y., Samuel B.S., Breen P.C., Ruvkun G. (2014). Caenorhabditis elegans pathways that surveil and defend mitochondria. Nature.

[42] Levine B., Kroemer G. (2008). Autophagy in the pathogenesis of disease. Cell.

[43] Longatti A., Tooze S.A. (2009). Vesicular trafficking and autophagosome formation. Cell Death Differ..

[44] Zhu Y., Casey P.J., Kumar A.P., Pervaiz S. (2013). Deciphering the signaling networks underlying simvastatin-induced apoptosis in human cancer cells: Evidence for non-canonical activation of RhoA and Rac1 GTPases. Cell Death Dis..

[45] Hutagalung A.H., Novick P.J. (2011). Role of Rab GTPases in membrane traffic and cell physiology. Physiol. Rev..

[46] Sciarretta S., Zhai P., Shao D., Maejima Y., Robbins J., Volpe M., Condorelli G., Sadoshima J. (2012). Rheb is a critical regulator of autophagy during myocardial ischemia: Pathophysiological implications in obesity and metabolic syndrome. Circulation.

[47] Ravikumar B., Futter M., Jahreiss L., Korolchuk V.I., Lichtenberg M., Luo S., Massey D.C., Menzies F.M., Narayanan U., Renna M. (2009). Mammalian macroautophagy at a glance. J. Cell Sci..

[48] Hall M.N. (2008). mTOR-what does it do?. Transplant. Proc..

[49] Bartolomé A., Kimura-Koyanagi M., Asahara S., Guillén C., Inoue H., Teruyama K., Shimizu S., Kanno A., García-Aguilar A., Koike M., Uchiyama Y. (2014). Pancreatic β-cell failure mediated by mTORC1 hyperactivity and autophagic impairment. Diabetes.

[50] Bodemann B.O., Orvedahl A., Cheng T., Ram R.R., Ou Y.H., Formstecher E., Maiti M., Hazelett C.C., Wauson E.M., Balakireva M., Camonis J.H. (2011). RalB and the exocyst mediate the cellular starvation response by direct activation of autophagosome assembly. Cell.

[51] Bento C.F., Puri C., Moreau K., Rubinsztein D.C. (2013). The role of membrane-trafficking small GTPases in the regulation of autophagy. J. Cell Sci..

[52] Jiang P., Mukthavaram R., Chao Y., Nomura N., Bharati I.S., Fogal V., Pastorino S., Teng D., Cong X., Pingle S.C., Kapoor S. (2014). *In vitro* and *in vivo* anticancer effects of mevalonate pathway modulation on human cancer cells. Br. J. Cancer.

[53] Araki M., Maeda M., Motojima K. (2012). Hydrophobic statins induce autophagy and cell death in human rhabdomyosarcoma cells by depleting geranylgeranyl diphosphate. Eur. J. Pharmacol..

[54] Wei Y.M., Li X., Xu M., Abais J.M., Chen Y., Riebling C.R., Boini K.M., Li P.L., Zhang Y. (2013). Enhancement of autophagy by simvastatin through inhibition of Rac1-mTOR signaling pathway in coronary arterial myocytes. Cell Physiol. Biochem..

[55] Stenson P.D., Mort M., Ball E.V., Shaw K., Phillips A., Cooper D.N. (2014). The Human Gene Mutation Database: Building a comprehensive mutation repository for clinical and molecular genetics, diagnostic testing and personalized genomic medicine. Hum. Genet..

[56] Simon A., Kremer H.P., Wevers R.A., Scheffer H., de Jong J.G., van der Meer J.W., Drenth J.P. (2004). Mevalonate kinase deficiency: Evidence for a phenotypic continuum. Neurology.

[57] Hoffmann G.F., Charpentier C., Mayatepek E., Mancini J., Leichsenring M., Gibson K.M., Divry P., Hrebicek M., Lehnert W., Sartor K. (1993). Clinical and biochemical phenotype in 11 patients with mevalonic aciduria. Pediatrics.

[58] Haas D., Hoffmann G.F. (2006). Mevalonate kinase deficiencies: From mevalonic aciduria to hyperimmunoglobulinemia D syndrome. Orphanet. J. Rare Dis..

[59] Mandey S.H., Kuijk L.M., Frenkel J., Waterham H.R. (2006). A role for geranylgeranylation in interleukin-1β secretion. Arthritis Rheum..

[60] Kuijk L.M., Mandey S.H., Schellens I., Waterham H.R., Rijkers G.T., Coffer P.J., Frenkel J. (2008). Statin synergizes with LPS to induce IL-1β release by THP-1 cells through activation of caspase-1. Mol. Immunol..

[61] Cailliez M., Garaix F., Rousset-Rouvière C., Bruno D., Kone-Paut I., Sarles J., Chabrol B., Tsimaratos M. (2006). Anakinra is safe and effective in controlling hyperimmunoglobulinaemia D syndrome-associated febrile crisis. J. Inherit. Metab. Dis..

[62] Bodar E.J., Kuijk L.M., Drenth J.P., van der Meer J.W., Simon A., Frenkel J. (2011). On-demand anakinra treatment is effective in mevalonate kinase deficiency. Ann. Rheum. Dis..

[63] Galeotti C., Meinzer U., Quartier P., Rossi-Semerano L., Bader-Meunier B., Pillet P., Koné-Paut I. (2012). Efficacy of interleukin-1-targeting drugs in mevalonate kinase deficiency. Rheumatology (Oxford).

[64] Pontillo A., Paoluzzi E., Crovella S. (2010). The inhibition of mevalonate pathway induces upregulation of NALP3 expression: New insight in the pathogenesis of mevalonate kinase deficiency. Eur. J. Hum. Genet..

[65] Lamkanfi M., Dixit V.M. (2009). The inflammasomes. PLoS Pathog..

[66] Martinon F., Mayor A., Tschopp J. (2009). The inflammasomes: Guardians of the body. Annu. Rev. Immunol..

[67] Deretic V., Saitoh T., Akira S. (2013). Autophagy in infection, inflammation and immunity. Nat. Rev. Immunol..

[68] Lee H.K., Lund J.M., Ramanathan B., Mizushima N., Iwasaki A. (2007). Autophagy-Dependent Viral Recognition by Plasmacytoid Dendritic Cells. Science.

[69] Paludan C., Schmid D., Landthaler M., Vockerodt M., Kube D., Tuschl T., Münz C. (2005). Endogenous MHC class II processing of a viral nuclear antigen after autophagy. Science.

[70] Blanchet F.P., Moris A., Nikolic D.S., Lehmann M., Cardinaud S., Stalder R., Garcia E., Dinkins C., Leuba F., Wu L. (2010). Human immunodeficiency virus-1 inhibition of immunoamphisomes in dendritic cells impairs early innate and adaptive immune responses. Immunity.

[71] Nedjic J., Aichinger M., Emmerich J., Mizushima N., Klein L. (2008). Autophagy in thymic epithelium shapes the T-cell repertoire and is essential for tolerance. Nature.

[72] Harris J., Hartman M., Roche C., Zeng S.G., O’Shea A., Sharp F.A., Lambe E.M., Creagh E.M., Golenbock D.T., Tschopp J. (2011). Autophagy Controls IL-1 Secretion by Targeting Pro-IL-1 for Degradation. J. Biol. Chem..

[73] Auron P.E., Webb A.C., Rosenwasser L.J., Mucci S.F., Rich A., Wolff S.M., Dinarello C.A. (1984). Nucleotide sequence of human monocyte interleukin 1 precursor cDNA. Proc. Natl. Acad. Sci. USA.

[74] Matsushima K., Taguchi M., Kovacs E.J., Young H.A., Oppenheim J.J. (1986). Intracellular localization of human monocyte associated interleukin 1 (IL 1) activity and release of biologically active IL 1 from monocytes by trypsin and plasmin. J. Immunol..

[75] Shi C.S., Shenderov K., Huang N.N., Kabat J., Abu-Asab M., Fitzgerald K.A., Sher A., Kehrl J.H. (2012). Activation of autophagy by inflammatory signals limits IL-1β production by targeting ubiquitinated inflammasomes for destruction. Nat. Immunol..

[76] Andrei C., Dazzi C., Lotti L., Torrisi M.R., Chimini G., Rubartelli A. (1999). The secretory route of the leaderless protein interleukin 1β involves exocytosis of endolysosome-related vesicles. Mol. Biol. Cell.

[77] Zhou R., Yazdi A.S., Menu P., Tschopp J. (2011). A role for mitochondria in NLRP3 inflammasome activation. Nature.

[78] Shimada K., Crother T.R., Karlin J., Dagvadorj J., Chiba N., Chen S., Ramanujan V.K., Wolf A.J., Vergnes L. (2012). Oxidized Mitochondrial DNA Activates the NLRP3 Inflammasome during Apoptosis. Immunity.

[79] Ding Z., Liu S., Wang X., Khaidakov M., Dai Y., Mehta J.L. (2013). Oxidant stress in mitochondrial DNA damage, autophagy and inflammation in atherosclerosis. Sci. Rep..

